# Recombinant Incretin-Secreting Microbe Improves Metabolic Dysfunction in High-Fat Diet Fed Rodents

**DOI:** 10.1038/s41598-017-14010-x

**Published:** 2017-10-19

**Authors:** Paul M. Ryan, Elaine Patterson, Robert M. Kent, Helena Stack, Paula M. O’Connor, Kiera Murphy, Veronica L. Peterson, Rupasri Mandal, David S. Wishart, Timothy G. Dinan, John F. Cryan, Randy J. Seeley, Catherine Stanton, R. Paul Ross

**Affiliations:** 1 Teagasc Food Research Centre, Moorepark, Fermoy, Co, Cork, Ireland; 2 School of Microbiology, University College Cork, Co, Cork, Ireland; 3 APC Microbiome Institute, University College Cork, Co, Cork, Ireland; 4 Department of Biological Sciences, Cork Institute of Technology, Co, Cork, Ireland; 5 Department of Neuroscience, University College Cork, Co, Cork, Ireland; 6 Department of Biological Sciences, University of Alberta, Edmonton, Alberta Canada; 7 Department of Computing Science, University of Alberta, Edmonton, Alberta Canada; 8 National Institute for Nanotechnology, Edmonton, Alberta Canada; 9 Department of Psychiatry, University College Cork, Co, Cork, Ireland; 10 Surgery Departments, University of Michigan, Ann Arbor, MI USA

## Abstract

The gut hormone glucagon-like peptide (GLP)-1 and its analogues represent a new generation of anti-diabetic drugs, which have also demonstrated propensity to modulate host lipid metabolism. Despite this, drugs of this nature are currently limited to intramuscular administration routes due to intestinal degradation. The aim of this study was to design a recombinant microbial delivery vector for a GLP-1 analogue and assess the efficacy of the therapeutic in improving host glucose, lipid and cholesterol metabolism in diet induced obese rodents. Diet-induced obese animals received either Lactobacillus paracasei NFBC 338 transformed to express a long-acting analogue of GLP-1 or the isogenic control microbe which solely harbored the pNZ44 plasmid. Short-term GLP-1 microbe intervention in rats reduced serum low-density lipoprotein cholesterol, triglycerides and triglyceride-rich lipoprotein cholesterol substantially. Conversely, extended GLP-1 microbe intervention improved glucose-dependent insulin secretion, glucose metabolism and cholesterol metabolism, compared to the high-fat control group. Interestingly, the microbe significantly attenuated the adiposity associated with the model and altered the serum lipidome, independently of GLP-1 secretion. These data indicate that recombinant incretin-secreting microbes may offer a novel and safe means of managing cholesterol metabolism and diet induced dyslipidaemia, as well as insulin sensitivity in metabolic dysfunction.

## Introduction

GLP-1 is a short conserved mammalian peptide produced by enteroendocrine L-cells that can stimulate insulin production to lower postprandial glucose levels in patients with type-2 diabetes (T2D)^1,2^. While GLP-1 has been known to interact with an array of additional systems – including the brain^3^, liver^4^ and stomach^5^ – its role in lipid metabolism and cardiovascular health has only recently emerged^6^. However, the incretin is rapidly cleaved and rendered inactive by circulating dipeptidyl peptidase (DPP)-4. As such, numerous drugs have explored extending the effects of GLP-1, either by synthetic stimulation of the GLP-1 receptor (GLP-1R) by DPP-4-resistant analogues (*e*.*g*. exendatide and lirglutitide) or direct inhibition of DPP-4 activity (*e*.*g*. gliptins).

GLP-1, GLP-1R agonists and DPP-4 inhibitors have all shown varying abilities to suppress enteric cell chylomicron production, made phenotypically evident by a reduction in circulating triglycerides and apolipoprotein B-48 (apoB-48)^7–9^. Chylomicrons are triglycerides-rich lipoprotein particles, which are formed in the endoplasmic reticulum of enterocytes and transport triglycerides to adipose, skeletal and cardiac tissues. These structures have now been identified as contributors to arterial cholesterol accumulation and consequently, are at the core of dyslipidaemia-induced atherosclerosis in obese and insulin resistant individuals^10^. While it is acknowledged that GLP-1 impacts upon host satiety and weight, factors which could credibly alter lipid metabolism, these studies indicate a direct effect on enteric GLP-1R as the central mechanism. Moreover, GLP-1R signaling has also been shown to correct endothelial dysfunction^11^, while also reducing blood pressure by activating atrial natriuretic peptide^12^.

The propensity of certain members of the gut microbiota to prevent or progress cardiovascular disease (CVD), as well as other aspects of metabolic health, is becoming increasingly apparent^13^. Intestinal bacteria constantly deliver metabolites, such as short chain fatty acids, which regulate host metabolism^14^. In recent years, studies have harnessed the potential of engineering bacteria as live cell factories for the production of desirable ‘designer’ bioactive molecules or drugs *in vivo*, with the aim of managing human health issues from allergies to obesity^15–18^. These treatments have been termed synthetic signalling therapeutics^19^. A recent study from Duan *et al*. investigating the use of such a therapeutic in a streptozotocin model of type-1 diabetes (T1D) demonstrated the ability of a probiotic transformed to express the full-length, inactive form of GLP-1 (_1–37_) to restore one third of the host insulin production^20^. The strain was found to interact with small intestinal crypt epithelial cells through the signalling molecule, transforming them into insulin secreting β-like cells. Further to this, the Wallenberg Laboratory for Cardiovascular & Metabolic Research recently performed a similar study with *Lactococcus lactis* secreting the same full length peptide in diet-induced obese mice^21^. Indeed, this study also reported encouraging results; increasing pancreatic insulin secretion and glucose clearance in a model of hyperglycaemia, thereby improving glucose tolerance.

The aim of the present study was to assess the potential of a long-lasting GLP-1 analogue (termed KGLP-1) secreting microbe to improve metabolic function in diet-induced obese rodents. More specifically, it was hypothesised that the microbe may attenuate insulin resistance, and the gross atherogenic dyslipidaemia associated with obesity and T2D. This study builds on previous research demonstrating the practicality of microbes as incretin-signalling vectors in models of metabolic dysfunction.

## Results

### GLP-1-Secreting Microbe Elicits Insulin Secretion in Murine β-Cells

**Figure 1:**
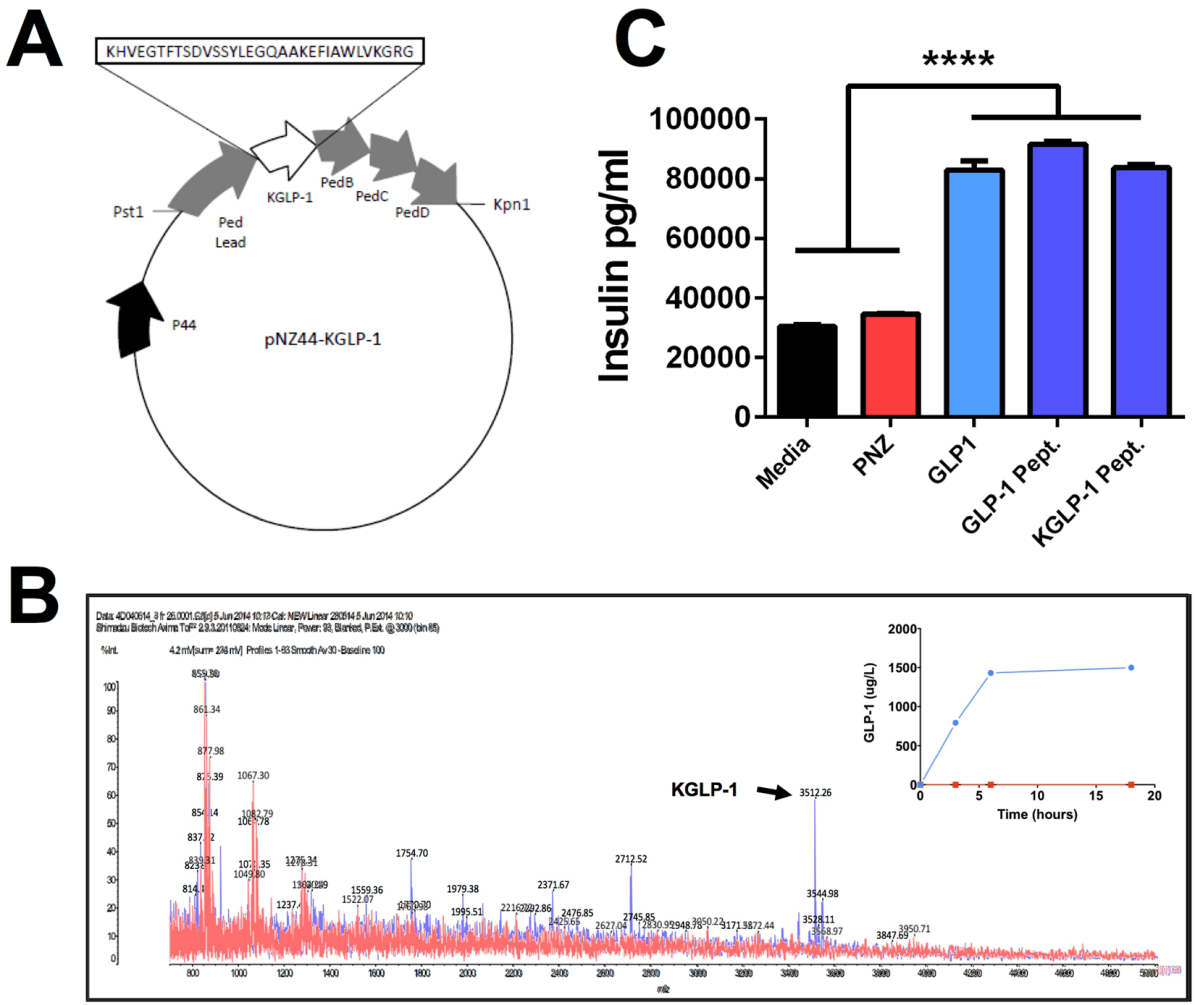
Microbial-Produced GLP-1 Displays Insulinotropic Activity Comparable to Synthetic Peptide. (**A**) pNZ44-KGLP-1 plasmid construct with KGLP-1 amino acid sequence; (**B**) Overlaid MALDI-TOF spectra of PNZ (red) and GLP1 (blue) spent cell-free growth medium HPLC fractions, with KGLP-1 peak (3512 Da) indicated by arrow. Inset graph displays KGLP-1 production during culture of PNZ (red) and GLP1 (blue) microbes, as assessed by ELISA of cell-free media; (**C**) Insulin production by Rinm5F-Beta cells treated with Lactobacillus paracasei NFBC 338 PNZ or GLP1 filtrate preparations, or pure KGLP-1 and GLP-1 peptides (50uM). Data was analysed by one-way ANOVA, significant differences are represented by ****(p < 0.0001) and plots depict replicates with mean and SEM.

*In vitro* assays revealed that synthetic KGLP-1 compared favourably with Sigma Standard GLP-1_7–37_ in terms of insulinotropic activity. Moreover, when compared with Sigma Standard GLP-1_7–37_ and synthetic KGLP-1 at levels of 50 µM the microbial-generated DPP-4-resistant GLP-1 induced similar levels of insulin secretion (Fig. 1C), which were higher than typical reported post-prandial levels of GLP-1 in circulation *in vivo* ^22^. In addition, PNZ supernatant demonstrated no significant insulinotropic effects, when compared to the control media.

### Experiment I – Short-Term Diet-Induced Obese Rat Model

#### Short-Term GLP-1-Secreting Microbe Intervention Improves Diet-Induced Dyslipidaemia

**Figure 2:**
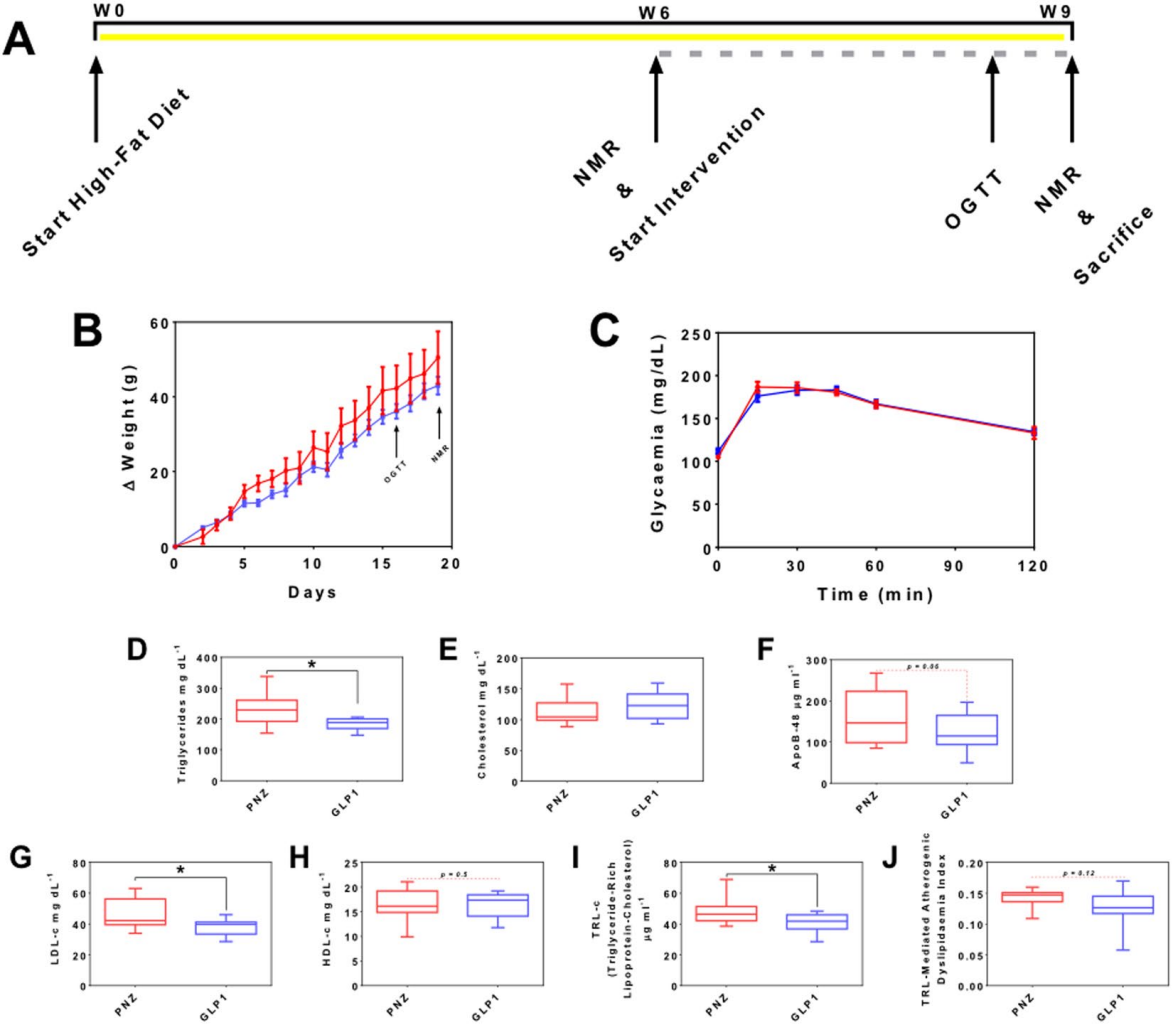
Short-Term GLP-1-Secreting Lactobacillus paracasei Intervention Modulates Lipid but Not Glucose Metabolism. Experiment I: (**A**) Schematic of study timeline, with high-fat diet represented by yellow bar and intervention by broken grey bar. (**B**) Weight gain of PNZ (red) and GLP1 (light blue) rats over treatment period. (**C**) Oral Glucose Tolerance Test: blood glucose of PNZ (red) and GLP1 (light blue) treatment groups before and after glucose challenge. (**D**) Fasting serum triglycerides, (**E**) total cholesterol, (**F**) apoB-48, (**G**) LDL-C and (**H**) HDL-C of PNZ (red) and GLP1 (light blue) treatment groups at cull for experiment I. (**I**) Triglyceride-rich lipoprotein cholesterol (TLR**-**C) and (**J**) triglyceride-rich lipoprotein-mediated atherogenic dyslipidaemia (TLR**-**AD) index were calculated from the data above. Figures represent the mean with SEM or max and min bars (n = 9). Significant differences are represented by *(p < 0.05).

While fasted serum lipid profiling revealed no significant alteration in total cholesterol, LDL-C was found to be significantly reduced in the group receiving GLP-1-secreting *Lb*. *paracasei* NCIMB 338 (GLP1; 19% reduction, *p < *0.05; Fig. 2G), while treatment with GLP1 also resulted in 20% lower serum triglycerides levels compared with the isogenic control (PNZ; *p < *0.05; Fig. 2D). Mean serum apoB-48 level in PNZ group was 164 µg.ml^−1^, while GLP1 rats demonstrated a mean of 122 µg.ml^−1^ (Fig. 2F); trending towards significance (*p* = 0.06). Finally, calculations applied to fasting lipid and cholesterol data uncovered a significant reduction in triglyceride-rich lipoprotein cholesterol (TRL-C) in the GLP1 group when compared to PNZ (*p < *0.05; Fig. 2I), while TRL-mediated atherogenic dyslipidaemia (TLR-AD) index was unchanged (Fig. 2J). Despite these alterations in lipid metabolism, no significant alteration was observed in rat weight gain (Fig. 2B). Although GLP1-treated rats displayed a corrected serum lipid and cholesterol profile, oral glucose tolerance test (OGTT) results suggest that they failed to regulate their blood glucose more effectively than the control rats (Fig. 2C). There were no significant differences between the two groups at any of the selected time points or in area under the curve (AUC), and there was no significant difference in IR index between the two groups (Supplementary Table S2). Finally, compositional sequencing of the caecum microbiome revealed no significant alterations (Supplementary Table S3 and Figure S1), indicating that any metabolic effect was a direct result of the GLP1 intervention on the host, and not microbiome-mediated.

### Experiment II – Extended Diet-Induced Obese Mouse Model

#### Extended *Lactobacillus paracasei* NFBC 338 Intervention Attenuates Adiposity Independently of GLP-1-Expression

**Figure 3:**
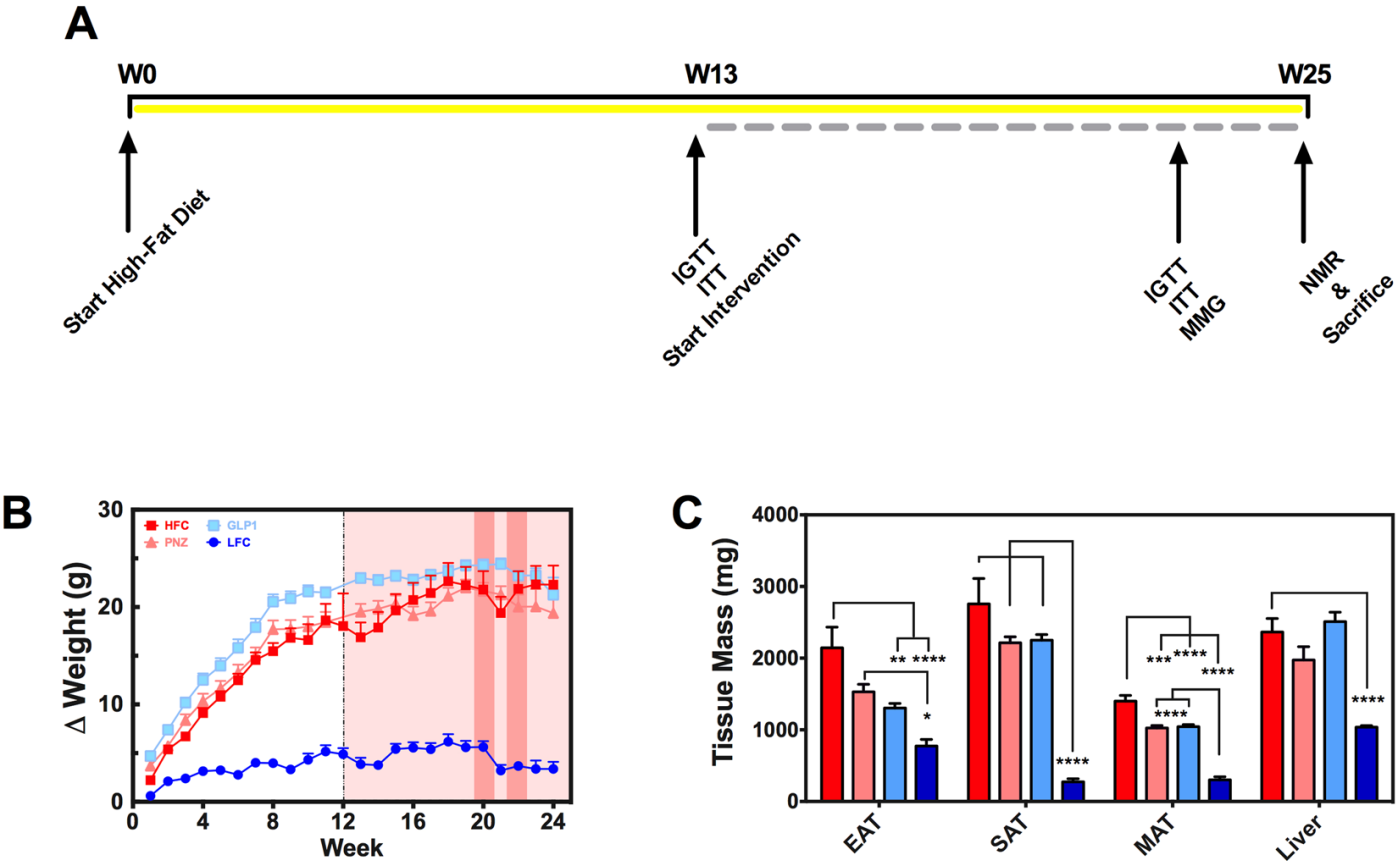
Lactobacillus paracasei Attenuates Mouse Adiposity Independently of GLP-1-Expression. Experiment II: (**A**) Mouse trial design and procedures detailed above, with high-fat diet represented by yellow bar and intervention by broken grey bar. (**B**) Delta change in body weights over the pre-feeding (white background) and intervention (red background) periods for HFC (dark red; n = 13), PNZ (light red; n = 14), GLP1 (light blue; n = 14) and LFC (dark blue; n = 14). Tall dark red boxes represent metabolic test weeks (IPGTT/ITT and mixed meal gavage, respectively). (**C**) Epididymal (EAT), subcutaneous (SAT) and mesenteric adipose tissue (MAT), and liver tissue weights are also depicted. Data was analysed by one-way ANOVA with Bonferroni correction, significant differences are represented by *(p < 0.05), **(p < 0.01), ***(p < 0.001), ****(p < 0.0001), where the asterisks are coloured according to the group with which a significant difference was recorded. Plots depict replicates with mean and SEM.

Over the 12 week intervention period (Fig. 3A), body weights of the high-fat diet fed groups remained similar (Fig. 3B). However, both microbial interventions (PNZ and GLP1) accumulated significantly less mesenteric adipose tissue (MAT; *p* < 0.001) and significantly reduced epididymal adipose tissue (EAT) when compared to the HFC (*p* < 0.05; Fig. 3C). Despite such promising effects, GLP1 treated mice were consistently found to be statistically similar to PNZ mice in each of these metrics, suggesting that the expression of GLP-1 was not the functional component for reduction of adiposity. Rather, this is likely the result of *Lb*. *paracasei* NFBC 388 or alternative metabolites derived from the microbe.

#### Extended GLP-1-Secreting Microbe Intervention Improves Glucose Metabolism and Insulin Sensitivity

**Figure 4:**
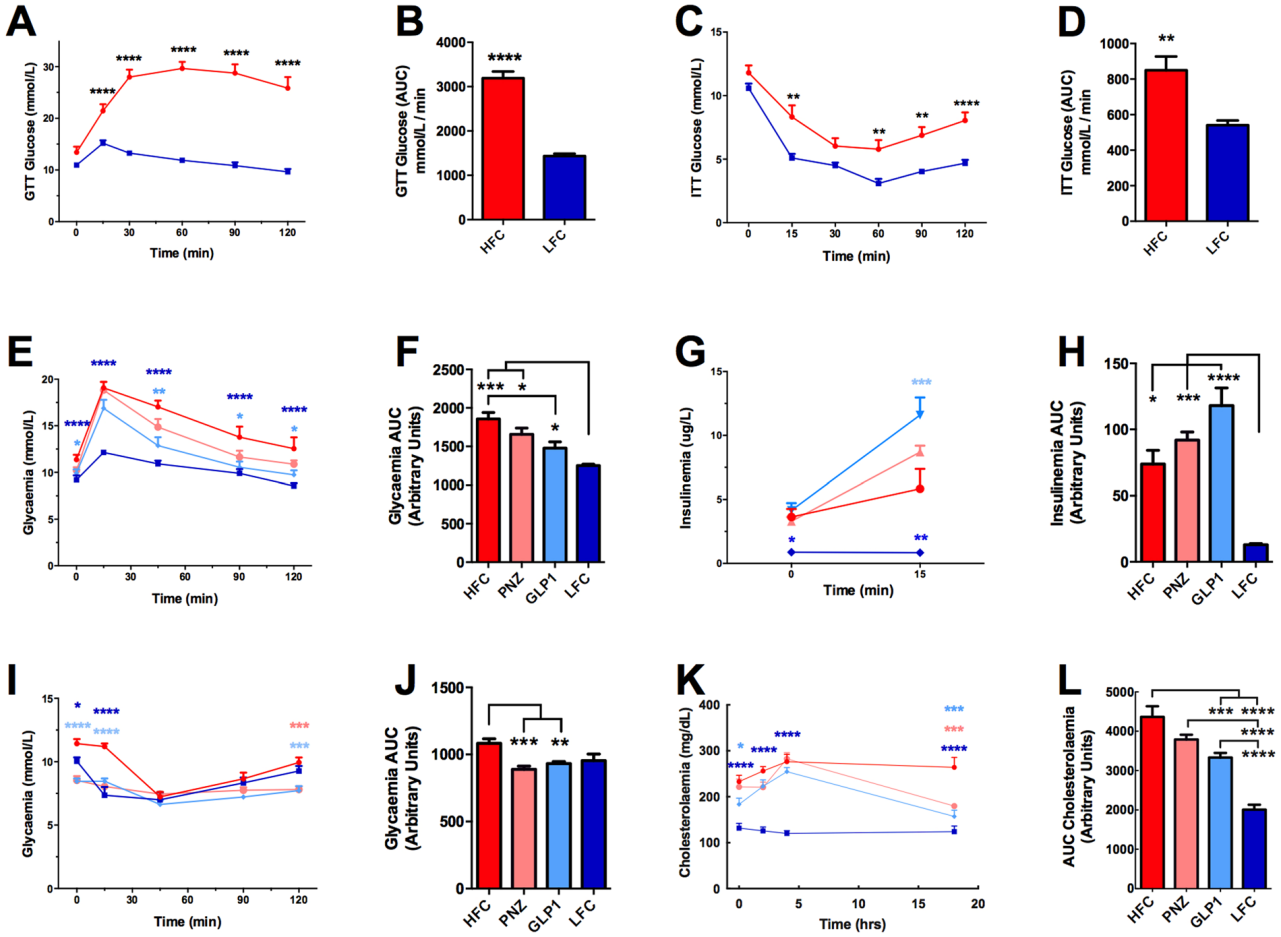
GLP-1-Expressing Lactobacillus paracasei Improves Glucose & Cholesterol Metabolism While Promoting Glucose-Dependant Insulin Secretion. Experiment II: HFC (n = 7) and LFC (n = 7) animals were assessed for 1 g/kg glucose (IPGTT) and 0.75 IU/kg insulin (ITT) tolerance at week 12 of feeding, prior to commencement of interventions. (**A**) IPGTT; (**B**) IPGTT area under the curve (AUC); (**C**) ITT; (**D**) ITT AUC. At week 22 of feeding/week 10 of intervention HFC (n = 7), PNZ (n = 14), GLP1 (n = 13) and LFC (n = 7) animals were again assessed for glucose and insulin tolerance. (**E**) IPGTT glucose levels; (**F**) IPGTT glucose AUC; (**G**) IPGTT insulin levels (T0 and T15); (**H**) IPGTT insulin AUC; (**I**) ITT glucose levels; (**J**) ITT glucose AUC. Animals were assessed for lipid and cholesterol metabolism by oral gavage with a complete meal (**K,L**) Ensure Plus, Abbott Nutrition). Data was analysed by one-way ANOVA with Bonferroni correction. Significant differences are represented by *(p < 0.05), **(p < 0.01), ***(p < 0.001), ****(p < 0.0001), where the asterisks are coloured according to the group which they represent. Plots depict replicates with mean and SEM.

Following 13 weeks of high-fat diet prefeeding (Fig. 3A), glucose and insulin tolerance tests revealed that HF-fed mice were sufficiently intolerant when compared to LF-fed animals to commence interventions (Fig. 4A–D). Following an additional 12 weeks feeding in conjunction with intervention, mice were again assessed for their glucose (Fig. 4E,F) and insulin tolerance (Fig. 4G,H). Prior to the glucose challenge, solely GLP1 (*p* < 0.05) and LFC (*p* < 0.0001) mice displayed a reduced fasting blood glucose concentration when compared to the HFC (Fig. 4E). The results of the intraperitoneal glucose tolerance test (IPGTT) assay demonstrate that extended intervention with GLP1 significantly attenuates the disruption of glucose metabolism observed in HF-fed mice, with reduced blood glucose at 0 min (*p* < 0.05), 45 min (*p* < 0.01), 90 min (*p* < 0.05) and 120 min (*p* < 0.05; Fig. 4E), while PNZ treatment had no such effect. Further to this, GLP1 mouse AUC glycaemia was found to be significantly lower than the HFC (*p* < 0.05), while PNZ was not (Fig. 4F). However, neither GLP1 glycaemia nor glycaemia AUC were found to be significantly lower at any time point, when compared to the PNZ group.

Bloods were also drawn to assess insulin concentration at 0 min (fasting) and 15 min of the IPGTT. Interestingly, while all high-fat fed groups were statistically similar prior to IP, at 15 min GLP1 mice displayed a significantly elevated insulin level when compared to the HFC (*p* < 0.01; Fig. 4G), which resulted in an elevated insulinaemia AUC (*p* < 0.05; Fig. 4H). This result is particularly validating for the therapy, as GLP-1-based interventions exert insulinotropic effects only in the presence of glucose. This in turn corroborates the *in vitro* data demonstrating the insulinotropic effects of the secreted KGLP-1 peptide. Curiously, both GLP1 (*p* < 0.0001) and PNZ intervention (*p* < 0.0001) reduced blood glucose levels in the first two time points of the insulin tolerance test (ITT; Fig. 4I). In addition, both groups again appeared to significantly reduce ITT AUC when compared to the HFC (*p* < 0.01). However, the great deal of variation observed in the ITT may detract from the reliability of this metric.

#### Extended GLP-1-Secreting Microbe Intervention Improves Cholesterol Metabolism

Assessment of serum prior to mixed meal gavage indicates that, of the two treatment groups, solely the GLP1 group demonstrated reduced fasting cholesterol (*p* < 0.05; Fig. 4K,L). To determine how capable the mice were of managing their post-prandial cholesterol, we delivered the complete liquid meal orally. Following the challenge, the HFC group cholesterol levels spiked high and remained at the same concentration (~270 mg/dL) over the 18 h monitoring period. Although the two other HF-fed groups reacted in a similar fashion over the first 4 h, they both displayed a greatly reduced serum cholesterol concentration at 18 h post-feed (*p* < 0.001). Importantly, although PNZ-treated mice displayed a reduced AUC (*p* < 0.05), GLP1 cholesterolemia AUC was found to be further reduced when compared to that of PNZ (*p* < 0.01).

#### Extended *Lactobacillus paracasei* NFBC 338 Intervention Alters Host Amino Acid, Biogenic Amine and Phosphotidylcholine Metabolism

**Figure 5:**
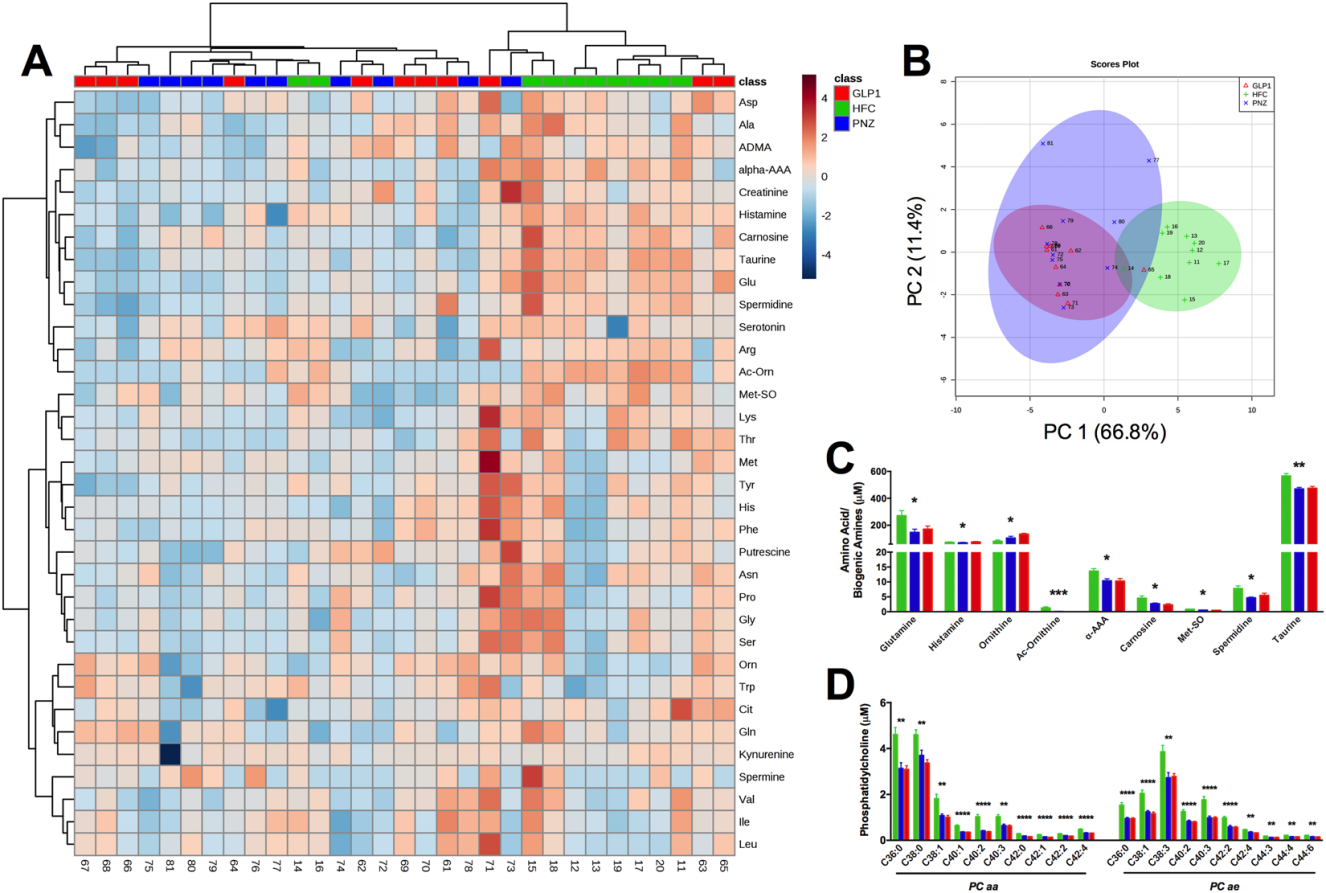
Lactobacillus paracasei Modulates Host Amino Acid, Biogenic Amine & Phosphotidylcholine Metabolism Independently of GLP-1-Expression. (**A**) Experiment II: Serum metabolome heatmap, with dendogram clustering according to sample likeness. (**B**) Principle Coordinate Analysis (PCoA) plot displays HFC (green), PNZ (.blue) and GLP1 (red) samples. (**C,D**) Quantitative data is displayed for the metabolites which were significantly altered by GLP1 or PNZ; this includes amino acids and biogenic amines, as well as diacyl-phosphatidylcholine (PC aa) and acyl-alkyl-phosphatidylcholine (PC ae). *(q < 0.05), **(q < 0.01), ***(q < 0.001), ****(q < 0.0001) represent significant differences between both PNZ/GLP1 and HFC. Plots depict individual replicates (n = 10) with mean and SEM.

Targeted analysis of the fasted mouse serum metabolome unveiled a number of key metabolic alterations as a result of microbial intervention. Figure 5A depicts a sample × metabolite heatmap which displays samples in a dendogramatic alignment. Interestingly, we see that the HFC serum metabolomes appear to cluster away from those of both PNZ and GLP1 groups, both of which remain intertwined. This microbial-modulated shift in the metabolome is further portrayed in the amino acid and biogenic amine principle coordinate analysis (PCoA) plot (Fig. 5B), as both PNZ and GLP1 cluster clearly away from the HFC; but again the two microbial groups remain intertwined. Exaggeration of this clustering can be seen in guided sparse partial least square-discriminate analysis (sPLS-DA) plot loadings (Supplementary Figure S3A and B). The specific fluctuations observed include increased levels of ornithine (*q* < 0.05), and decreased levels of amino acids glutamine and histamine (*q* < 0.05), and several biogenic amines (ac-ornithine [*q* < 0.001], α-AAA [*q* < 0.05], carnitine [*q* < 0.05], met-SO [*q* < 0.05], spermidine [*q* < 0.05] and taurine [*q* < 0.01]) in PNZ/GLP1 when compared to the HFC (Fig. 5C). In addition, a plethora of long-chain diacyl PC (PC aa) and acyl-alkyl PC (PC ae) reductions were also observed in the microbial intervention groups when compared to the HFC, and this shifted the serum molecular signature considerably (Fig. 5D). However, the secretion of GLP-1 had no additional effect on the metabolic signature.

## Discussion

The management of metabolic syndrome and related conditions must be of top priority in global health agenda. An estimated 382 million people worldwide are currently living with diabetes^23^, meaning that the development of affordable and effective means of managing the symptoms of the disease, particularly in the early stages, is becoming increasingly urgent. GLP-1-based therapies have shown their efficacy in improving glucose metabolism in T2D^3^. However, due to the degradability of short peptides^24^, these treatments are often limited to intramuscular or intraperitoneal injection. The use of bacteria as synthetic signalling therapeutics for the management or prevention of certain disease states has garnered attention, but has shown limited success until recently. One such therapeutic which has demonstrated considerable potential, and is now in phase 2a clinical trials, is an interleukin-10 secreting *L*. *lactis*, which has previously been shown to protect against colitis in a preclinical model of the disease^25^.

As introduced previously, the works of both Duan *et al*.^20^ and Arora *et al*.^21^ act as a proof of concept for incretin-secreting bacterial therapies, demonstrating that recombinant microbes may have applications in modulation of metabolic dysfunction. Further to this, the work described herein details specifically the action of an active GLP-1 analogue, and also extrapolates the utility of such therapies in the management of the atherogenic dyslipidaemia associated with obesity and diabetes. In addition, we should address the specific compositions and bioactivities of the secreted peptides. Indeed, the full length precursor form of GLP-1 has shown the potential to reach circulation and activate pancreatic β-cells; however, its half-life is still very short due to DPP-4 activity, perhaps necessitating relatively regular dosing. Conversely, assuming similar peptide expression and absorption rates between the microbes and hosts in each of these studies, the recombinant *Lb*. *paracasei* in the present study may in fact be more suitable as a therapeutic considering it secretes a DPP-4-resistant analogue of active GLP-1, thereby demonstrating greatly increased half-life.

While commercial GLP-1-based therapies have been shown to have a corrective effect on atherogenic dyslipidaemia and hypertriglyceridemia^8,26^, until recently the mechanisms involved in this effect were poorly understood. However, it now appears that GLP-1-based therapies can have broader cardioprotective effects through two main pathways. Firstly, when GLP-1R located in the heart are stimulated, this causes the release of atrial natriuretic peptide, a hormone which acts in lowering blood pressure through smooth muscle relaxation^12^. Secondly, and perhaps most significantly with respect to our data, GLP-1 and GLP-1R agonists are known to act on enterocyte GLP-1R, resulting in a repression of chylomicron formation^3,27^. This in turn reduces circulating levels of triglycerides, apoB-48 and to some degree cholesterol^9,28^, which has important implications for CVD development. Furthermore, a reduction in pancreatic triglycerides, such as is seen following bariatric surgery, is associated with profound beneficial implications for T2D host metabolic health, in terms of insulin secretory abilities^29^.

In *experiment I*, fasting rat serum LDL-C and triglycerides levels in rats receiving the GLP-1-secreting probiotic were significantly reduced by ~20%, while apoB-48 levels were found to be approaching a significant reduction (*p* = 0.06). As has previously been alluded to, triglycerides and apoB-48 represent primary components of intestinally derived chylomicrons. These results indicate that the GLP-1-secreting probiotic has the potential to reduce the abundance of these potentially atherogenic fragments, perhaps through the previously identified enterocyte GLP-1R pathway. In order to assess the CVD risk in the rats following the three week intervention, the *log*(TG)/HDL-C calculation for TRL-AD was applied to the data. In addition, TRL-C calculations were applied to evaluate the levels of intestine-derived chylomicron and hepatic-derived very-low density lipoprotein. While TRL-C showed significant improvement as a result of GLP1 treatment, TRL-AD was found to be statistically similar between the two groups. It is likely that the three week intervention period was too short to demonstrate a more complete correctional effect on host atherogenic dyslipidaemia state. Entirely promising is the complementary reduction in fasting cholesterol and overall an improvement in postprandial cholesterol metabolism was observed following GLP1 intervention in a separate model organism, under the modified conditions detailed in *experiment II*. It is important to note that humans spend the majority of their day in postprandial-state^27^ and, as a result, fed-state cholesterol and lipid profiles have been determined to be more indicative of CVD risk^30,31^.

Despite there being no significant effect on glucose metabolism in the rat study (*experiment I*), the GLP1 intervention did have a substantial effect on this metabolic aspect in the diet-induced obese (DIO) mice in *experiment II*. This may have been a result of chronic rather than acute intervention, and establishment of the diet-induced phenotype prior to intervention. The substantial increase in the diet fat proportion (60% in *experiment II* versus 40% in *experiment I*) also likely contributed to the increased effect size in this test. In addition, IPGTT have been shown to produce different results when compared to OGTT^12^. However, it is clear from the GTT and ITT results in *experiment II* that *Lb*. *paracasei* intervention had some degree of effect on glucose homeostasis, independently of GLP-1 expression. Therefore, it is plausible that a similar effect may indeed have been observed in *experiment I*, had a HFC group been included. The same effect can be seen in attenuation of mouse adiposity observed in *experiment II*, all of which suggest that the naïve bacteria must secrete additional unidentified factors which contribute somewhat to host metabolic function.

Finally, investigation into the serum metabolome revealed a range of alterations in amino acids and lipids, with previously reported implications for host metabolism. PC, a family of phospholipids which contain a choline headgroup, are key structural constituents of apoB, and are therefore integral components of chylomicron, LDL-C and VLDL-C particles. In turn, it has been previously demonstrated that impaired hepatic PC synthesis leads to reduced circulating levels of these particles, brought about by depleted synthesis and elevated uptake. Therefore, it is likely that the extensive reduction in PC is, at least in part, responsible for the shifts in both cholesterol and triglyceride metabolism observed for microbial-intervention groups. Interestingly, these shifts in lipid metabolism were previously found to be coupled with reduced atherogenesis. While metabolomics research is still, in many ways, in catologuing stages and the full implications of such metabolites for host metabolic function are not entirely clear, links are being identified between certain PC and metabolic dysfunction. In line with this, a number of long-chain PC have been shown to be elevated in clinical studies of T2D, correlating negatively with insulin secretion and sensitivity^32^. While the manner in which microbes interact with host lipid metabolism have been explored previously in preclinical models^33,34^, the exact molecular underpinnings remain incompletely elucidated.

## Conclusion

This study is the first to assess the application of a recombineered microbe as a synthetic signalling therapeutic for the delivery of an active and long-lasting analogue of GLP-1 to the host intestine. The expression system used utilized the leader and export machinery of the bacteriocin pediocin to enable efficient production and export of GLP-1. The aim of this intervention was to improve glucose and lipid-load handling in DIO rodent models of metabolic dysfunction. The results indicate that three weeks of GLP1 intervention was sufficient to reduce fasted rat serum triglycerides and LDL-C levels substantially. In addition, GLP1 intervention significantly improved overall cholesterol metabolism and reduced fasting hypercholesterolemia in DIO mice, when compared to the non-incretin-secreting isogenic control. However, extended intervention with the GLP-1-expressing microbe was necessary to improve host glucose metabolism. It is likely that the lipid metabolism effects observed were mediated by intestinal GLP-1R activation, while correction of glucose homeostasis may be the result of pancreatic beta-cell GLP-1R activation. Finally, these results indicate that GLP-1-secretion was in fact not necessary for attenuation of adiposity and alteration of the serum amino acid and lipid metabolome by *Lb*. *paracasei* NFBC 338 over a 12-week intervention period. These data indicate that recombinant, hormone-secreting microbes may offer a novel therapeutic avenue for the management of host metabolic dysfunction. In conclusion, we build upon previous proof-of-concept work, which has proven the utility of GLP-1-secreting microbes in preclinical models of T1D and T2D, by demonstrating an effect on lipid metabolism and atherogenic lipid profile, while also profiling the effects of microbial therapy on the host metabolome.

## Materials and Methods

### Recombinant Microbe

The nucleotide sequence for KGLP-1 was synthesised by overlap extension PCR (OE-PCR) using primers 1, 2, 3 and 4 outlined in Supplementary Table S1. DNA was extracted from *Pediococcus acidilacti* DPC 5492 and the pediocin leader (*pedlead*) sequence was amplified using primers 5 and 6. A splicing OE-PCR reaction using primers 2 and 5 was used to combine the KGLP-1 sequence (Fig. 1A) and pediocin leader. PCR reactions were carried out at 60 °C using KOD polymerase (Merck Millipore). Mature pediocin was generated using primers 6 and 7. The *pedB*, (specifies immunity), *pedC* and *pedD* genes (membrane bound proteins required for peptide secretion)^35^ were amplified from *P*. *acidlilacti* DPC 5492 DNA using primers 7 and 8. The restriction sites *Pst1* and *Kpn1* were incorporated into the sequence to allow incorporation into the plasmid pNZ44 via ligation reaction^36^. Restriction polymerases and T4 DNA ligase were purchased from New England Biolabs (240 County Rd., Ipswich, MA, USA).

Plasmid constructs were transformed into competent *E*. *coli* which was spread plated on LB agar containing 10 µg/ml chloramphenicol and incubated overnight. Successful transformants were treated with a Plasmid Mini kit (Qiagen). Electrocompetent *Lb*. *paracasei* NFBC 338 cells were prepared using 3.5X SMEB (1 M sucrose, 3.5 mM MgCL2). Plasmids were electroporated into *Lb*. *paracasei* NFBC 338 which was plated as described earlier with the addition of 10 µg/ml chloramphenicol. The relevant DNA fragments were verified by restriction endonucleases and gel electrophoresis. Correct DNA sequences were validated by DNA sequencing (Beckman Coulter Genomics, Essex CM22 6TA, United Kingdom). *Lb*. *paracasei* NFBC 338 is an established probiotic strain which has demonstrated amenability for recombineering and is resistant to the stresses of gastrointestinal transit^37^. The recombinant KGLP-1-secreting variant was designated as GLP1 and the isogenic control for this construct was *Lb*. *paracasei* NFBC 338 solely containing the pNZ44 plasmid, transformed in the same manner and was subsequently designated PNZ.

All bacteria were routinely cultured on de Mann Rogosa Sharp (MRS; Difco) anaerobically at 37 °C with the addition of 10 µg ml^−1^ chloramphenicol (MRS_CM10_; Sigma Aldrich, Ireland) to select for recombinant bacteria. Bacteria were firstly cultured from −80 °C stock on solid agar media and all subsequent subcultures were in broth.

### KGLP-1 Peptide Detection

Peptide expression and secretion was confirmed by matrix-assisted laser desorption ionization time of flight mass spectrometry (MALDI-TOF MS; Fig. 1B) of a high performance liquid chromatography fraction of the cell-free spent MRS broth. In brief, spent MRS culture broth was centrifuged at 10,000 x *g* for 10 min, before being passed through a 0.22 μm filter. Samples were then passed through a 3 ml, 200 mg, C18 SPE pre-equilibrated with methanol and water. The column was washed with 3 ml 30% ethanol and then 2 ml 70% isopropan-2-ol, 0.1% TFA (IPA). The IPA was removed from the sample and it was applied to an analytical Proteo Jupiter RP-HPLC column running a 27–55% gradient over 35 minutes where buffer B is 90% acetonitrile 0.1% TFA. Previously a 25–55% gradient with buffer B as 100% acetonitrile 0.1% TFA was used had to reduce the acetonitrile concentration of buffer B to extend the life of the degasser on the HPLC, consequently KGLP will elute slightly later than previously. Fractions were collected and fractions 31–34 from each run were checked for the presence of the 3512 Da KGLP-1 using MALDI TOF mass spectrometry.

In addition, the kinetics of microbial KGLP-1 secretion were analysed in cell-free spent MRS broth during culture at several points over an 18 hour period by total GLP-1 MSD kit (Meso Scale Discovery; Fig. 1B *inset*), as per the manufacturer’s instructions.

### *In Vitro* Insulinotropic Activity Assay

RINm5F Insulinoma cells (*Rattus norvegicus*) (ATCC-CRL-11605) (LGC Standards, Queens Road, Teddington, Middlesex, TW11 0LY, England) were cultured in RPMI-1640 (LGC Standards) medium containing 10% (v/v) foetal bovine serum, and 1% (v/v) Penicillin-Streptomycin solution (Sigma Aldrich: Cat. No. P0781). All cells were maintained in 75 cm2 sterile tissue culture flasks (Corning Inc, NY, USA) at 37 °C in an atmosphere of 5% CO_2_. Cells were passaged when the confluency of the flasks was approximately 90%. For insulinotrophic studies, cells were treated with trypsin and seeded into six well tissue culture plates (Sarstedt, Sinnottstown Lane, Drinagh, Co. Wexford, Ireland) at a density of 1 × 10^5^ cells/well.

Insulinotrophic properties of *Lb*. *paracasei* NFBC 388 GLP1 generated peptides were investigated using a method previously described^38^ with minor alterations. Briefly 2 ml of RINm5F cells were cultured as described. Cell monolayers were washed using KREBS-Ringer bicarbonate buffer (Sigma Aldrich) and starved for 40 min at a basal glucose level via addition of 1 ml of KREBS buffer containing 1.1 mM glucose (Sigma Aldrich). Buffer was removed and the cells were stimulated with prepared (0.22 nm filtered) filtrate from *Lb*. *paracasei* NFBC 338 PNZ and GLP-1. Each sample was incubated with glucose at 2.2 mM, 4.4 mM or 8.8 mM for 20 min. Sigma GLP-1 and synthetic KGLP-1 were also assayed at 50 µmol, nmol and pmol for comparison. Following incubation samples were collected and treated with protease and phosphatase inhibitor (10 µl/ml) (Fisher Scientific UK Ltd, Bishop Meadow Road, Loughborough, UK.; Cat. No. PN78443) before being applied to a MSD insulin plate (Cat. No. K152BZC-1). Insulin production was measured according to manufacturer’s instructions using a MSD Sector 2400.

### Treatment Preparation

The engineered probiotic and control bacteria were cultured as previously described until stationary phase and centrifuged (16,900 x *g* for 15 min, at 4 °C; SLA-3000 rotor, Sorvall RC B5-Plus). The cell pellet was washed twice with phosphate buffered saline (PBS; Sigma Aldrich), resuspended at ~2 × 10^10^ CFU.ml^−1^ in sterile 15% trehalose (Sigma Aldrich) and 1 ml aliquots were dispensed into 2 ml lyophilisation vials. The vials were lyophilised on a 24 h program (freeze temperature −40 °C, additional freeze 1 min, condenser set point −60, vacuum set point 600 mTorr; VirTis AdVantage Wizard 2.0) and stored at 4 °C until use. Bacteria were resuspended in distilled water each day in order to deliver 10^9^ CFU/d to each animal. Gastric transit of the strains was assessed each week by culturing serial dilutions of fresh faecal samples on MRS_CM10_ plates, as described previously.

### Animal Experiments

All animals were acclimatised for 1 week in the housing facility prior to trial commencement. Animals were group-housed at 21 °C in a 12-h light–dark cycle (light cycle 07:00–19:00). Two animal experiments were conducted in this study. *Experiment I* was a diet-induced obesity (DIO) rat model carried out in the Metabolic Disease Institute of University of Cincinnati, approved by and performed according to the guidelines of the Institutional Animal Care and Use Committee of the University of Cincinnati. *Experiment II* was a DIO mouse model performed in the APC Microbiome Institute of University College Cork and experimental procedures were carried out as per the protocols approved by the University College Cork Ethics Committee, with a license acquired through the Health Products Regulatory Authority.

#### Experiment I

As an initial proof of concept study, 20 Long-Evans rats were maintained on a high-fat butter diet (4.54 kcal/g; 41% fat; Research Diets, NJ, USA) *ad libitum* for 6 weeks prior to treatment. The animals were individually caged and kept on a 12 h light/dark cycle. Following the initial 6 weeks, body composition (NMR; EchoMedical Systems, Houston, TX) and weights were assessed, allowing the animals to be distributed into either the treatment or control groups, counterbalancing for these parameters. Following allocation, the rats remained on the high-fat diet while concurrently receiving either the incretin-secreting *Lb*. *paracasei* (GLP1; *n* = 10) or the isogenic control strain which solely expressed the empty pNZ44 plasmid vector (PNZ; *n* = 10) for an additional 17 d prior to the oral glucose tolerance test (OGTT). Animals were culled following 21 d of intervention.

#### Experiment II

Three week old C57BL/6 mice were acquired from Envigo UK and maintained in a 12 h light/dark cycle and on a high-fat diet (Open Source Diets [D12492 – 60% kcal from fat; Research Diets Inc.]) *ad libitum* for 25 weeks in total and for the final 12 weeks exposed to 10^9^ CFU/mouse.d^−1^ of GLP1 (*n* = 14), PNZ (*n* = 14) or no intervention (HFC; *n* = 14). An additional group of animals were maintained on a low-fat control diet (LFC; *n* = 14; Open Source Diets [D15072701 – 10% kcal from fat and equal parts corn starch and sucrose; Research Diets Inc.]) concurrently, acting as a negative control for development of obesity and insulin resistance. Animal weight gain and food intake were continuously assessed. In addition, intraperitoneal glucose and insulin tolerance tests were performed at weeks 12 and 24, while a mixed-meal gavage test was also performed at the end of the intervention to explore lipid and cholesterol metabolism.

### Metabolic Assessment

#### Oral Glucose Tolerance Test

Rats from *experiment I* were fasted overnight prior to OGTT. Baseline blood glucose was recorded in duplicate at T0 by tail bleed (Accu-Chek glucometers and strips; Roche, UK) and 2 g/kg 25% (w/v) dextrose was delivered intragastrically by oral gavage. Blood glucose was subsequently recorded in the same manner at 15, 30, 45, 60 and 120 min post glucose loading. In addition, β-cell function was assessed by insulin resistance index following quantification of insulin (Rat Insulin ELISA, Mercodia) in serum bleeds at 0 and 15 min. The equation used to calculate insulin resistance (IR) index was as follows^39^:

1 $$IR\,index=Glucose\,area\,under\,the\,curve(AUC)\ast Insulin\,AUC\ast {10}^{-4}$$

#### Intraperitoneal Glucose Tolerance Test

Mice from *experiment II* were fasted from the start of light-cycle for 6 hours prior to IPGTT. Baseline blood glucose was recorded in duplicate at T0 by tail bleed (Accu-Chek glucometers and strips; Roche) and 1 g D-glucose per kg of bodyweight was delivered by intraperitoneal injection. Blood glucose was subsequently recorded in the same manner at 15, 45, 90 and 120 min post glucose loading. In addition, β-cell function was assessed by IR index following quantification of insulin (Mouse Insulin ELISA, Mercodia) in plasma bleeds 0 and 15 min. The equation used to calculate IR index was as above.

#### Insulin Tolerance Test

ITT were performed on fasted mice from *experiment II* in the same manner as the IPGTT, with intraperitoneal injection of 0.75 IU insulin per kg of bodyweight. Blood glucose was monitored by the same method at T0 and the same timepoints post insulin challenge.

#### Mixed Meal Gavage

Mice from *experiment II* were fasted from the start of light-cycle for 6 hours prior to mixed meal gavage. Each mouse received 200 µl Ensure Plus (Abbott Nutrition, Ireland) by gastric oral gavage, and tail bleeds were performed at T0, 2, 4 and 18 h post gavage. The plasma was then analysed for cholesterol levels (EnzyChrom colorimetric assay; Cambridge Biosciences, UK).

### Serum Lipid and ApoB-48

Total serum cholesterol (TC) levels were determined in duplicate as per the manufacturer’s instructions using the EnzyChrom colorimetric assay (ECCH-100, BioAssay Systems, Hayward, CA, USA). Similarly, serum high-density lipoprotein-cholesterol (HDL-C), low-density lipoprotein cholesterol (LDL-C) and triglycerides were determined by their respective LabAssay assays (Wako Diagnostics, Germany). ApoB-48 in rat serum was quantified by ELISA (MyBioSource, SD, USA). Finally, the equation validated by Hermans *et al*.^40^ was applied to calculate TRL-C, and is as follows:

2 $$\begin{array}{rcl}TRL-C & = & TC-[(0.0106\ast TC-0.0036\ast triglycerides\\  &  & +\,0.017\ast [0.65\ast (TC-HDL-C)+6.3]\\  &  & -\,0.27)\ast 38.6]-HDL-C\end{array}$$

TLR-AD index was assessed by the following formula^41^:

3 $$TRL-AD=log[TG]/HDL-C$$

### Compositional Microbiome Sequencing

At the end of *Experiment I*, caecum content bacterial DNA was assessed to ensure that the effects observed were not mediated by fluctuations in the microbiome composition. Roughly 200 mg of caecal content was weighed out and added to a 2 ml tube containing sterile silicon beads. Extraction buffer was added and the tubes were vortexed for 3 min each. Following this, microbial DNA was extracted using the QIAamp DNA Stool Mini Kit protocol (Qiagen, UK). The primer pair 5′-TCGT CGGC AGCG TCAG ATGT GTAT AAGA GACA GCCT ACGG GNGG CWGC AG-3′ and 5′-GTCT CGTG GGCT CGGA GATG TGTA TAAG AGAC AGGA CTAC HVGG GTAT CTAA TCC-3′ were used to amplify the V3-V4 regions of the 16 S genes found in the isolated microbial DNA, as per the preparation manual for the Illumina MiSeq platform. Illumina Nextera kit (Illumina Nextera XT, Illumina) was used to tag samples with unique barcodes prior to quantification with the Qubit High Sensitivity DNA kit (Life Technologies, Ireland), pooling and sequencing.

FLASH (FLASH: fast length adjustment of short reads to improve genome assemblies) was applied for the assembly of resulting 300 bp paired-end reads. Additional sequence read processing, which included quality filtering based on a quality score of > 25 and removal of mismatched barcodes and sequences below length thresholds, was performed within QIIME (version 1.8.0). USEARCH (version 7, 64-bit) was utilised for denoising, chimera detection and clustering into operational taxonomic units (OTUs) (97% identity). OTU sequences were subsequently aligned using PyNAST (PyNAST: python nearest alignment space termination), after which taxonomy was assigned at 97% similarity against the SILVA SSURef database release 111. Alpha diversity estimates and beta diversity were calculated using QIIME and PCoA plots were created in EMPeror (version 0.9.3-dev; Emperor) and used to visualise differences in beta diversity based on UniFrac distances.

### Serum Metabolome Profiling

Serum collected from mice at cull of *Experiment II* was analyzed using the Biocrates AbsoluteIDQ p180 Kit (BIOCRATES Life Sciences AG, Austria). Following initial extraction and derivatization steps, a range of specific amino acids, biogenic amines, acylcarnitines, lysophosphotidylcholines, phosphotidylcholines (PC), sphingomyelins and hexoses present in the samples were detected and quantified on an ABI 4000 Q-Trap mass spectrometer (MDS Sciex) run in conjunction with a reverse-phase HPLC-column.

### Primary Splenocyte Stimulation

Spleens were removed immediately subsequent cull at *Experiment II* and maintained on chilled RPMI media prior to mechanical disruption and filtration through 70 μm Nylon Cell Strainers to isolate a single cell suspension. Red blood cells were removed following the procedure detailed in the Mouse Erythrocyte Lysis solution (Sigma Aldrich). Isolated cells were washed twice, counted by trypan blue exclusion and resuspended in DMEM supplemented with 10% foetal bovine serum (FBS) and 1% penicillin/streptomycin antibiotics to a concentration of 2 × 10^6^ cells/ml. Wells were stimulated with 1 μg/ml lipopolysaccharide^42^ (Sigma Aldrich) and incubated at 37 °C in 5% CO_2_ for 48 hours, while duplicate wells were also left unstimulated as controls. Plates were then centrifuged at 300 g and supernatants aspirated and stored at for analysis. Supernatant concentrations of the cytokines interleukin (IL)-10, IL-6 and tumour necrosis factor (TNF)-α were assessed using the mouse V-PLEX Plus Proinflammatory Panel 1 Kit (K15048G-1; Meso Scale Discovery, Ireland).

### Serum ELISA and Enzymatic Assays

Trunk serum was analysed by ELISA for levels of insulin (Mercodia Mouse Insulin ELISA; Cat No. 10-1247-01; Uppsala, Sweden), leptin (Crystal Chem Inc.; Cat. No. 90030; IL 60515, USA), C-peptide (Crystal Chem Inc.; Cat. No. 90050; IL 60515, USA), adiponectin (Crystal Chem Inc.; Cat. No. 80569; IL 60515, USA) and glucagon (Mercodia Glucagon ELISA – 10 μl; Cat No. 10-1281-01; Uppsala, Sweden). Trunk serum was analysed by enzymatic assay for glucose (Crystal Chem Inc.; Cat. No. 81692; IL 60515, USA) and glycated haemoglobin (HbA1c, Crystal Chem Inc.; Cat. No. 80310; IL 60515, USA) according to the protocols described by the manufacturers.

### Statistical Analysis

All datasets, apart from microbiome and metabolome data, were analysed in Prism 5 (GraphPad Software Inc.) by one-way analysis of variance (ANOVA) or unpaired, two-way Student’s t-test when stated, both with Bonferroni correction for multiple testing. Metabolomic data was log normalised prior to ANOVA and multivariate analyses in the MetaboAnalyst metabolomics analysis suite^43^. Sparse partial least square discriminant analysis (sPLS-DA) was also performed on metabolomic data^44^. Finally, non-normal sequencing data were analysed by Kuskal-Wallis, followed by 2-tailed Mann-Whitney U pairwise comparisons, with Bonferroni correction for multiple testing. For metabolomic and microbiome data, a false discovery rate (FDR) *q* value less than 0.05 was considered statistically significant.

### Data Availability

Sequencing and metabolomics data is to be made freely available in a public database and all additional *in vitro* and animal data is available upon request.

## Electronic supplementary material

Dataset 1
