# Author Correction: Recombinant Incretin-Secreting Microbe Improves Metabolic Dysfunction in High-Fat Diet Fed Rodents

**DOI:** 10.1038/s41598-020-59133-w

**Published:** 2020-02-06

**Authors:** Paul M. Ryan, Elaine Patterson, Robert M. Kent, Helena Stack, Paula M. O’Connor, Kiera Murphy, Veronica L. Peterson, Rupasri Mandal, David S. Wishart, Timothy G. Dinan, John F. Cryan, Randy J. Seeley, Catherine Stanton, R. Paul Ross

**Affiliations:** 1 Teagasc Food Research Centre, Moorepark, Fermoy, Co, Cork, Ireland; 2 School of Microbiology, University College Cork, Co, Cork, Ireland; 3 APC Microbiome Institute, University College Cork, Co, Cork, Ireland; 4 Department of Biological Sciences, Cork Institute of Technology, Co, Cork, Ireland; 5 Department of Neuroscience, University College Cork, Co, Cork, Ireland; 6 Department of Biological Sciences, University of Alberta, Edmonton, Alberta Canada; 7 Department of Computing Science, University of Alberta, Edmonton, Alberta Canada; 8 National Institute for Nanotechnology, Edmonton, Alberta Canada; 9 Department of Psychiatry, University College Cork, Co, Cork, Ireland; 10 Surgery Departments, University of Michigan, Ann Arbor, MI USA

Correction to: *Scientific Reports* 10.1038/s41598-017-14010-x, published online 19 October 2017

This Article contains errors in the Methods section under subheading ‘KGLP-1 Peptide Detection’. The following sentence was included erroneously:

“Previously a 25–55% gradient with buffer B as 100% acetonitrile 0.1% TFA was used had to reduce the acetonitrile concentration of buffer B to extend the life of the degasser on the HPLC, consequently KGLP will elute slightly later than previously.”

In addition, this article contains errors in the Methods section under the subheading ‘Primary Splenocyte Stimulation’.

“Plates were then centrifuged at 300 g and supernatants aspirated and stored at for analysis.”

should read:

“Plates were then centrifuged at 300 g and supernatants aspirated and stored at −80 °C for analysis.”

