# Supplementary material for: Recombinant Incretin-Secreting Microbe Improves Metabolic Dysfunction in High-Fat Diet Fed Rodents
Source: Sci Rep. 2017 Oct 19;7:13523. doi: 10.1038/s41598-017-14010-x (PMC5648875; doi:10.1038/s41598-017-14010-x)
Supplement: Supplementary file 1 — Dataset 1 [file 41598_2017_14010_MOESM1_ESM.doc]

**SUPPLEMENTARY DATA**

**Recombinant Incretin-Secreting Microbe Improves Metabolic Dysfunction in High-Fat Diet Fed Rodents**

Paul M Ryan ^1,2,3^, Elaine Patterson ^1,3^, Robert M Kent ^1,2^, Helena Stack ^4^, Paula M O’Connor ^1,2^, Kiera Murphy ^1^, Veronica L Peterson ^5^, Rupasri Mandal ^6^, David S Wishart ^6,7,8^, Timothy G Dinan ^3,9^, John F Cryan ^3,5^, Randy J Seeley ^10^, Catherine Stanton ^1,3^ & R Paul Ross^1,3,*^

*^1^ Teagasc Food Research Centre, Moorepark, Fermoy, Co. Cork, Ireland*

*^2^ School of Microbiology, University College Cork, Co. Cork, Ireland*

*^3^ APC Microbiome Institute, University College Cork, Co. Cork, Ireland*

*^4^ Department of Biological Sciences, Cork Institute of Technology, Co. Cork, Ireland*

*^5^ Department of Neuroscience, University College Cork, Co. Cork, Ireland*

*^6^ Department of Biological Sciences, University of Alberta, Edmonton, Alberta, Canada*

*^7^ Department of Computing Science, University of Alberta, Edmonton, Alberta, Canada*

*^8^ National Institute for Nanotechnology, Edmonton, Alberta, Canada*

*^9^ Department of Psychiatry, University College Cork, Co. Cork, Ireland*

*^10^ Surgery Departments, University of Michigan, Ann Arbor, MI USA*

**Supplementary Table S1 | *Relevant Primers for Molecular Work*. *Oligonucleotides used in plasmid construct generation.***

| ***Primer number*** | ***Nucleotide sequence 5'-3'*** |
| --- | --- |
| ***1*** | GGCCAATATCATTGGTGGTAAGCATGTGGAAGGCACCTTTACCAGCGATGTGAGCAGCTATCTGGAAGGCCAGGCGGC |
| ***2*** | CCGCTCGAGTTAGCCGCGGCCTTTCACCAGCCACGCAATAAATTCTTTCGCCGCCTGGCCTTCCAGATAGCTGCTCAC |
| ***3*** | AATCGGCGCCGGAAAG |
| ***4*** | TTCGTACACCTTCCGTGG |
| ***5*** | ACATCCATGGAAGAAGAAGGAGATTTTTG |
| ***6*** | GCACCTGCAGGCTAGCATTTATGATTAC |
| ***7*** | GCACCTGCAGCATTATGCTGAGCTGGCATC |
| ***8*** | GCACGGTACCGCGAGGATTTCACGG |

|  | **Body Composition** | | | | | | **Glucose Metabolism** | | | | | |
| --- | --- | --- | --- | --- | --- | --- | --- | --- | --- | --- | --- | --- |
|  | **Lean**  *(%)* | | **Fat**  *(%)* | | **Weight**  *(g)* | | **Glucose** | | **Insulin** | | **IR Index** | |
|  |  |  |  |  |  |  | *(mg dL^-1^)* | | *(µg L^-1^)* | |  |  |
| **PNZ** | 44.49 | 1.7 | 15.83 | 3.01 | 559.46 | 57.45 | 105 | 8.23 | 1.62 | 0.7 | 76.39 | 31.65 |
| **GLP1** | 44.00 | 2.2 | 16.22 | 3.37 | 560.04 | 33.32 | 111.6 | 10.5 | 1.30 | 0.92 | 62.30 | 35.14 |
| Data shown is mean with standard deviation. Glucose metabolism metrics were obtained from fasted serum.  No significant alterations were observed. | | | | | | | | | | | | |

**Supplementary Table S2 | *Experiment I* Rat Metabolic Parameters.** *Rat endpoint body composition with fasted glucose, insulin and insulin resistance index (glucose area under the curve x insulin area under the curve x 10^-4^).*

**Supplementary Table S3 | Phylum-Level 16S Compositional Sequencing.** *Experiment I rat caecum content 16S compositional sequencing relative abundances of the phyla detected. Data represents the mean with standard deviation (SD).* *No significant alterations were observed.*

|  | **GLP1** | | **PNZ** | |
| --- | --- | --- | --- | --- |
|  | **Mean** | **SD** | **Mean** | **SD** |
| Actinobacteria | 0.83361 | 1.24458 | 0.93625 | 1.01706 |
| Bacteroidetes | 26.12917 | 14.46262 | 25.61938 | 13.56603 |
| Candidate division TM7 | 0.00306 | 0.00827 | 0 | 0 |
| Cyanobacteria | 0.04194 | 0.06132 | 0.03813 | 0.09 |
| Deferribacteres | 0.29472 | 0.40156 | 0.35188 | 0.99323 |
| Deinococcus-Thermus | 0.00139 | 0.00182 | 0.00469 | 0.01039 |
| Firmicutes | 47.54444 | 16.47162 | 47.87781 | 14.15209 |
| Fusobacteria | 0 | 0 | 0.00063 | 0.00177 |
| Lentisphaerae | 0.00111 | 0.00182 | 0.00031 | 0.00088 |
| Proteobacteria | 2.29222 | 1.3062 | 2.03094 | 1.05231 |
| Tenericutes | 0.02861 | 0.04381 | 0.07844 | 0.10311 |
| Verrucomicrobia | 22.82972 | 16.45698 | 23.06156 | 14.54451 |


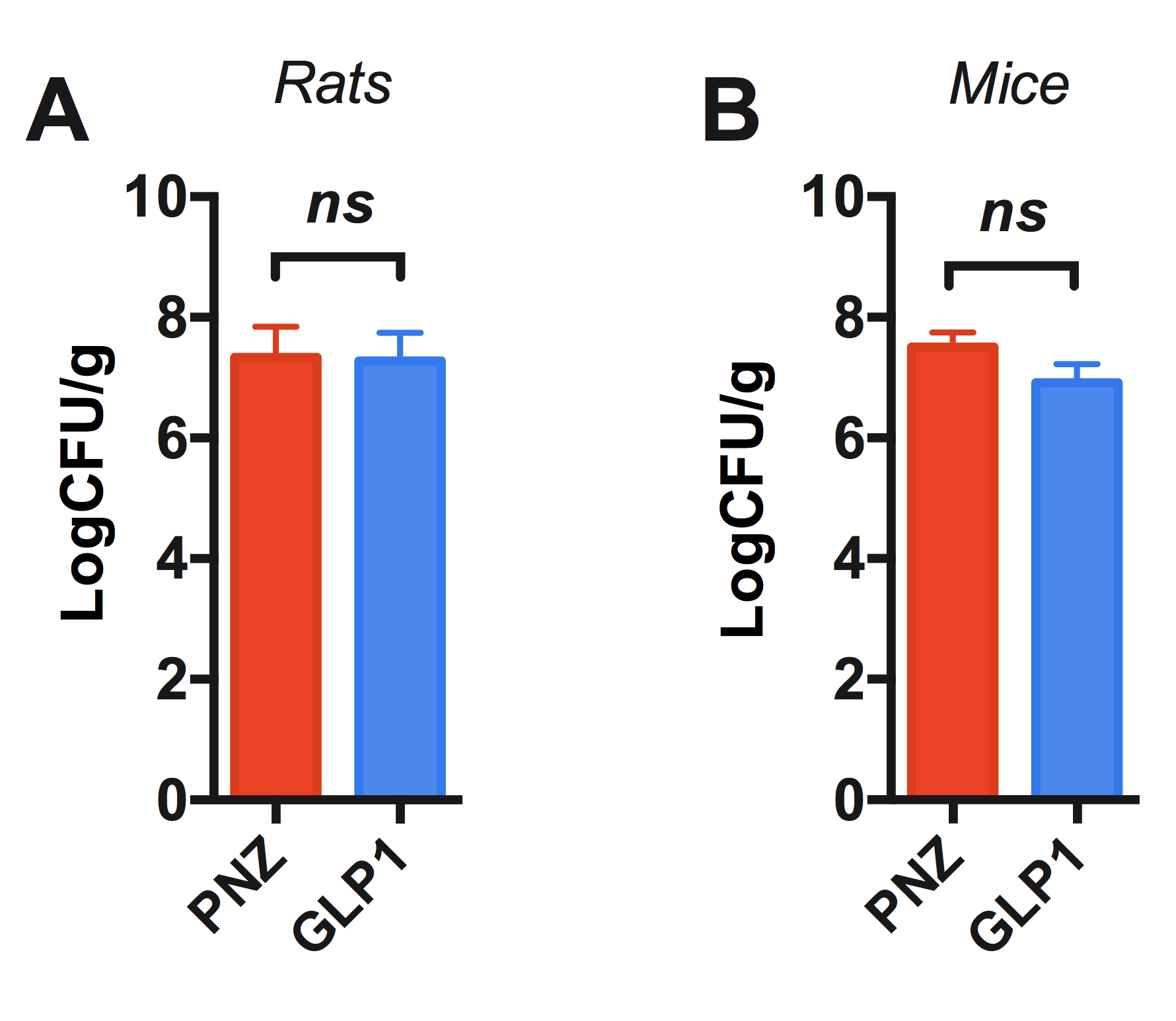


**Supplementary Figure S1 | Microbial Transit of Gastrointestinal Tract.** *Faecal load of PNZ (red) and GLP1 (blue) recombinant microbes in Experiment I (A) and Experiment II (B).* *Plots depict replicates with mean and SEM. Data was analysed two way t-test.*


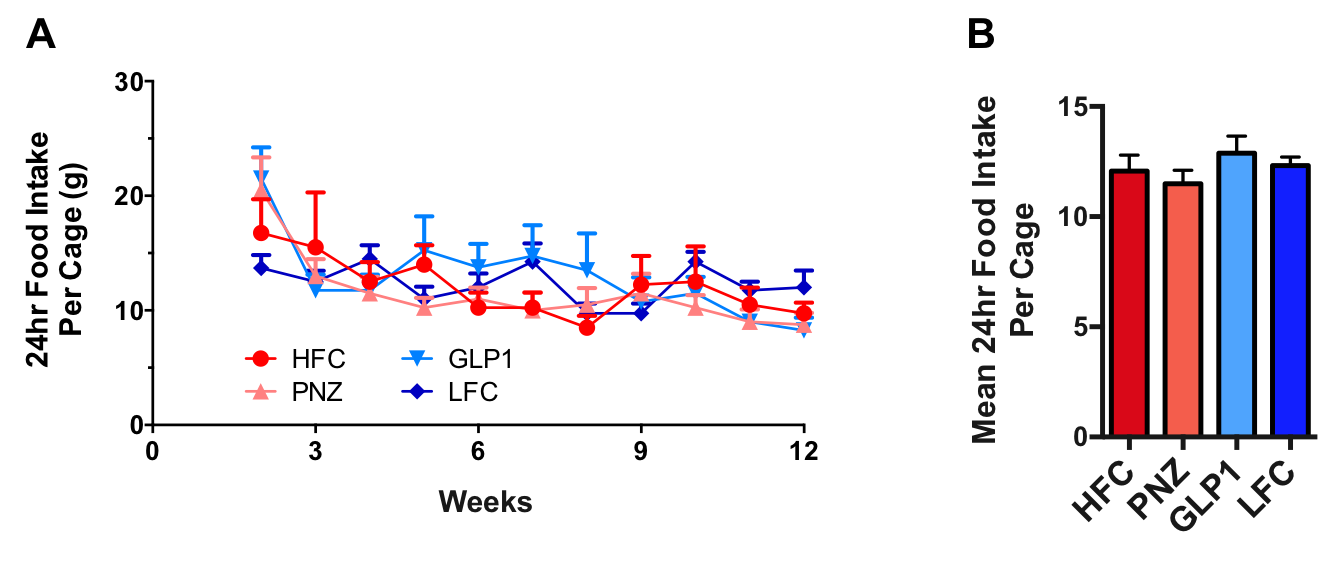


**Supplementary Figure S2 | Food Intake.** *(A) Food intake was assessed weekly for each cage within each group and the mean is displayed for each week. (B) The overall mean daily food intake was also calculated. Plots depict replicates (n = 13/14) with mean and SEM. Data was analysed by one way ANOVA with Bonferroni correction for multiple testing.*


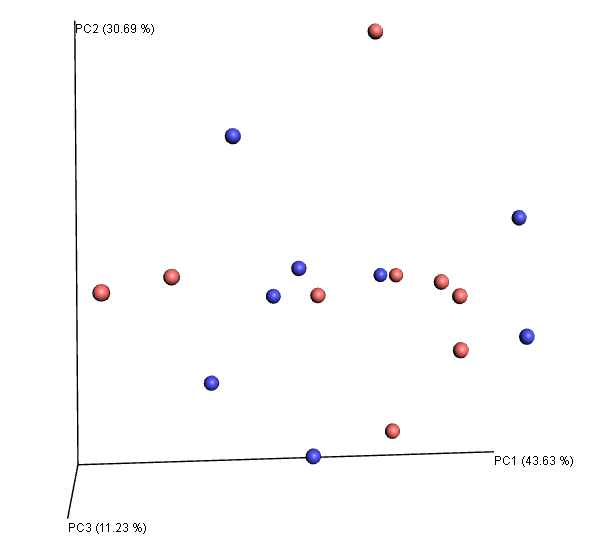


**Supplementary Figure S3 | Weighted Unfrac Principle Coordinates Analysis.** *Experiment I: Rat caecum content 16S compositional sequencing. Red points represent PNZ mice, while blue depict GLP1 mice.*

*
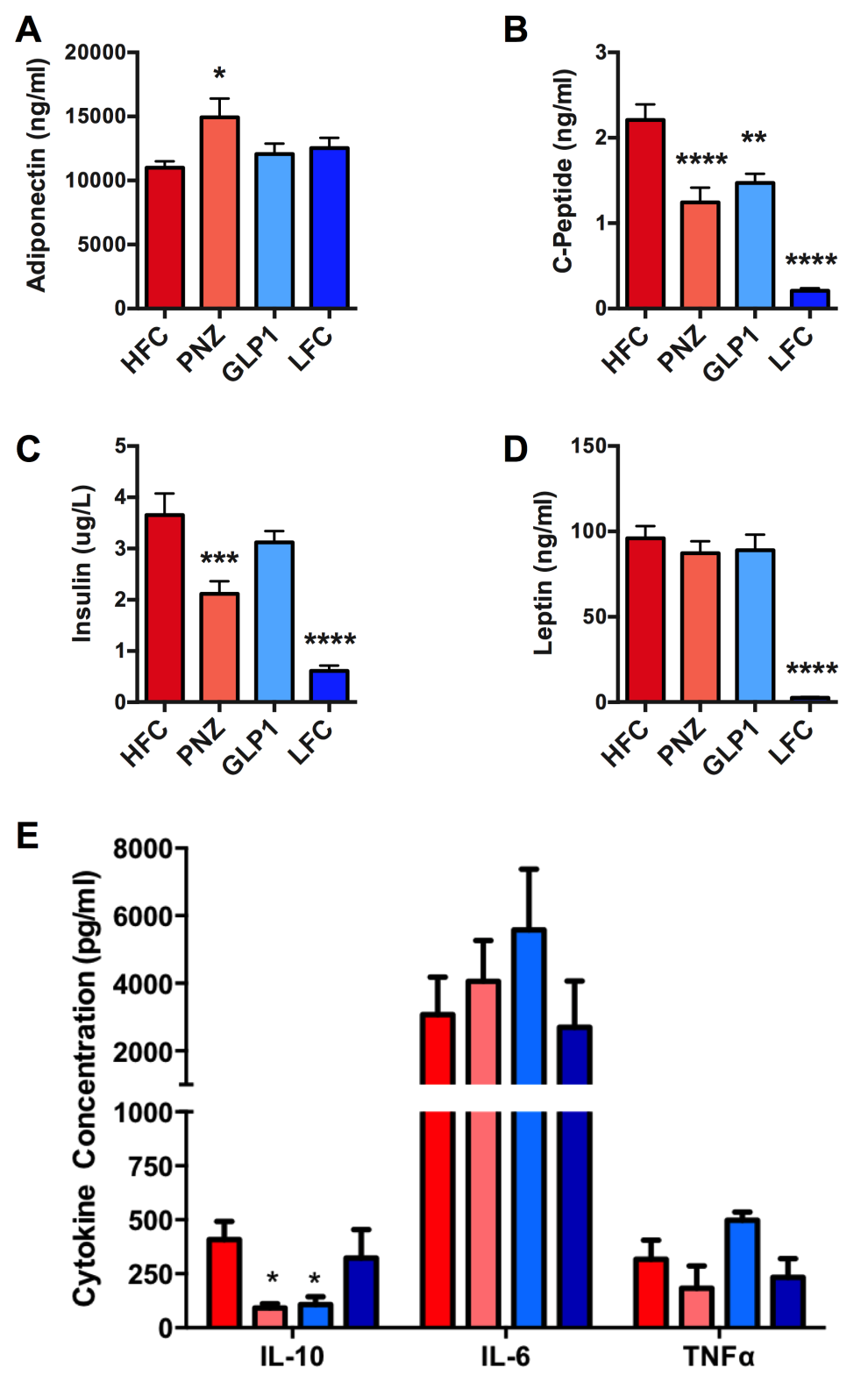
*

**Supplementary Figure S4 | Fasted Metabolic Markers & Cytokine Profile of Stimulated Spenocyte.** *Experiment II: Fasted levels of adiponectin (A), C-peptide (B), insulin (C) and leptin (D), as assayed by ELISA, are portrayed above. Pro and anti-inflammatory cytokine profiles of cultured primary splenocytes stimulated with lipopolysaccharide (E). HFC (dark red), PNZ (light red), GLP1 (light blue) and LFC (dark blue).* *Data was analysed by one-way ANOVA, significant differences are represented by * (p < 0.05), ** (p < 0.01), *** (p < 0.001) or **** (p < 0.0001), and plots depict replicates with mean and SEM.*

**
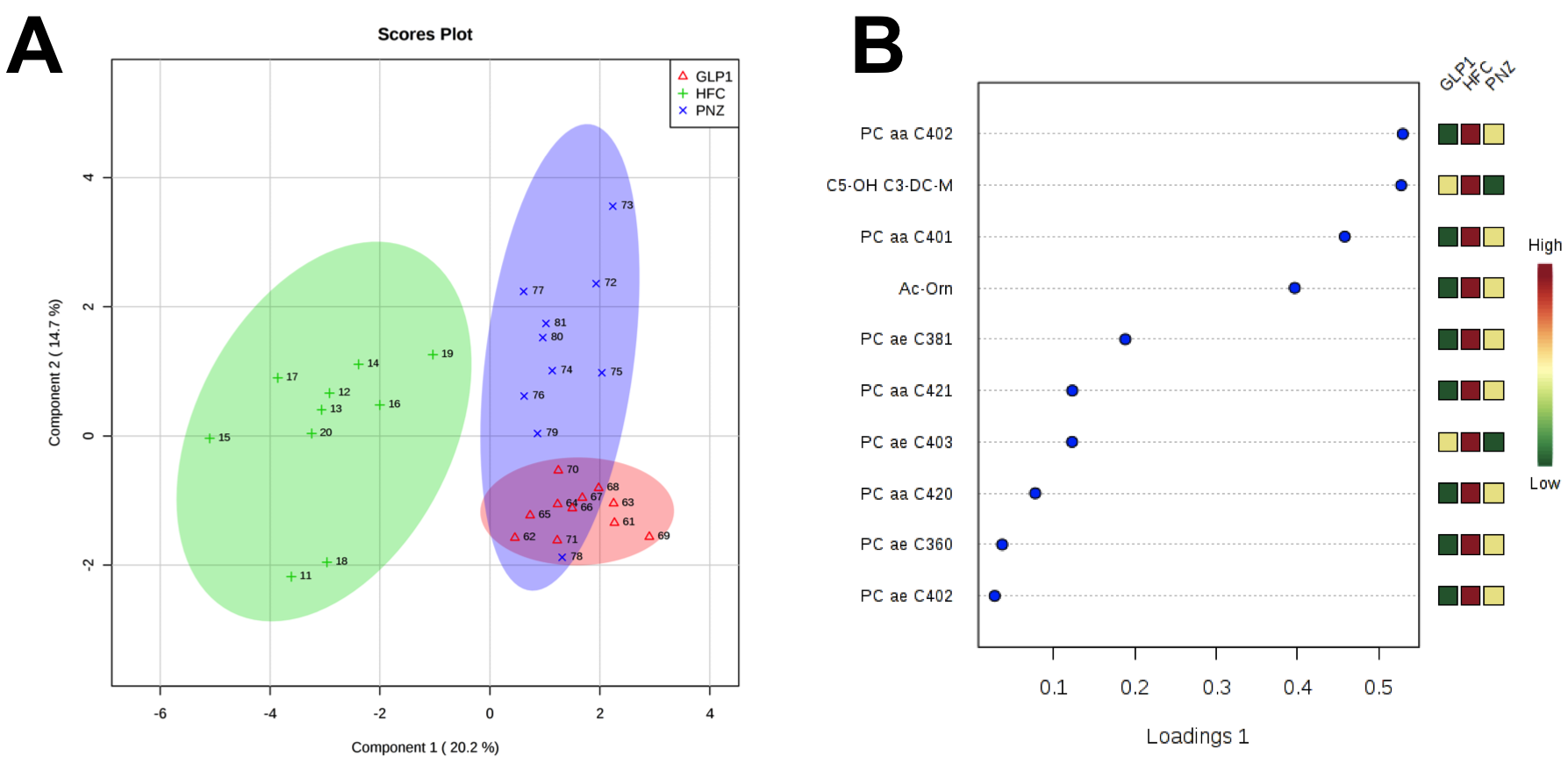
**

**Supplementary Figure S5 | Serum Metabolome Sparse Partial Least Square Discriminant Analysis & Loadings.** *Experiment II: Serum metabolome sparse PLS-DA plot (A) displays HFC (green), PNZ (.blue) and GLP1 (red) samples, with loading plot depicting metabolites of importance to projection (B).*
